# Semi-automated water sampling module for repeated sampling and concentration of *Bacillus cereus* group spores

**DOI:** 10.1038/s41598-023-27900-0

**Published:** 2023-01-16

**Authors:** Walid M. Hassen, Jonathan Vermette, Houman Moteshareie, Azam F. Tayabali, Jan J. Dubowski

**Affiliations:** 1 Laboratory for Quantum Semiconductors and Photon-based BioNanotechnology, Department of Electrical and Computer Engineering, Interdisciplinary Institute for Technological Innovation (3IT), CNRS IRL-3463, Université de Sherbrooke, 3000, boul. de l’Université, Sherbrooke, QC J1K 0A5 Canada; 2 Biotechnology Laboratory, Environmental Health Science and Research Bureau, Healthy Environments and Consumer Safety Branch, Health Canada, 251 Sir Frederick Banting Driveway, Ottawa, ON, K1A 0K9 Canada

**Keywords:** Environmental biotechnology, Laboratory techniques and procedures, Sensors and biosensors

## Abstract

Monitoring the presence of pathogenic *Bacillus* spores is important for industrial applications, as well as necessary for ensuring human health. *Bacillus thuringiensis* is used as a biopesticide against several insect pests. *Bacillus cereus* spores are a significant cause of food poisoning, and *Bacillus anthracis* is a recognized biosecurity threat. Laboratory-based methods, such as polymerase chain reaction, enzyme-linked immunosorbent assay, or matrix-assisted laser desorption ionization spectroscopy provide sensitive detection of bacteria and spores, but the application of those methods for quasi-continuous environmental monitoring presents a significant challenge requiring frequent human intervention. To address this challenge, we developed a workstation for quasi-autonomous monitoring of water reservoirs for the presence of bacteria and spores, and designed and validated the functionality of a microprocessor-controlled module capable of repetitive collection and pre-concentration of spores in liquid samples tested with fiberglass (FG), polyether sulfone and polyvinylidene fluoride filters. The best results were obtained with FG filters delivering a 20× concentration of *B. thuringiensis* and *B. cereus* spores from saline suspensions. The successful 20× pre-concentration of *Bacillus* spores demonstrated with FG filters could be repeated up to 3 times when bleach decontamination is applied between filtrations. Taken together, our results demonstrate an attractive instrument suitable for semi-automated, quasi-continuous sampling and pre-processing of water samples for biosensing of bacterial spores originating from a complex environment.

## Introduction

Bacterial endospores are formed by strains of *Bacillus* during nutrient limitation or when conditions are unfavorable to support vegetative growth. Spores are a major concern to the food industry because of spoiling in addition to the pathogenic potential of *Bacillus cereus* producing toxins inducing diarrhea and dehydration^1^. Spores are extremely resistant and durable; they can remain metabolically inactive where their survival is protected by multiple factors including low core water content, low permeability of the inner membrane, in addition to genome protection by specific DNA binding proteins, as well as a proteinaceous multilayering that additionally enables them to resist harsh physicochemical and environmental conditions that would otherwise kill vegetative cells^2^. In particular, *B. cereus* is present in a wide range of environments, such as soils, sediments, dust, or plants^3^. *Bacillus cereus* spores have been detected in rain water at concentrations ranging from 10^1^ to 2 × 10^2^ CFU/mL, and at 10^5^ CFU/mL in soil^4^. *Bacillus thuringiensis* spores have been extensively used as biopesticides by the agriculture and forestry industries^5,6^, and direct application of these spores to rivers and lakes is used to control mosquitoes and blackflies^7^.

Collecting microorganisms from contaminated water constitutes the important first step towards detection and enumeration of target microorganisms. Conventional methods are based on vacuum filtration of manually collected water samples^8^. Examples of the filtration methods and type of filters investigated for this purpose are provided in Table 1. The reported concentration factors (CF) of microorganisms achieved with filtration are in the 5–586 range^9–12^. It is important to indicate that CF depend on the filtered material and the backwash volumes. As an example, CF = 666 could be obtained for the filtered volume of 100 L and the backwash of 150 mL if all microbes were recovered^10^. But, it is unlikely to recover 100% of the microbes, not to mention that collecting 100 L samples is highly unpractical for conducting aquasi-continuous analysis of environmental water samples.

**Table 1:** Examples of filtration methods and concentration factors in microbial sampling.

Filter material	Filtration mode	Micro-organisms	CF	CF_Max_	Method of evaluation	Automatization	References
Ceramic	Tangential	*E. coli*	Up to 500	500	Volumetric concentration	Yes	Zhang et al.^9^
Polysulfone	Ultrafiltration Polysulfone dialysis filter MWCO 30 KDA	*S. enterica* *B.* atrophaeus var. *globigii* endospores *MS2* bacteriophage *E1* bacteriophage *C. parvum*	426–486 326–353 560–566 246–360 526–586	666	PCR	No	Polaczyk et al.^10^
Polycarbonate	Membrane filtration	*L. pneumophila*	31–82	100	Fluorescence microscopy	No	Yamaguchi et al.^11^
Polyvinylidene fluoride	Membrane filtration	*L. pneumophila*	5–20	33	Culture	Yes	Moumanis et al.^12^

The presence of spores in water has typically been determined by enumeration of colony growth on nutrient agar following contact of filtration membranes^13^. Methodology for filtration and pre-concentration of spores intended for further processing and analysis has largely been missing in the literature. Our preliminary results to pre-concentrate *B. cereus* spores with a polyvinylidene difluoride (PVDF) filter-based system were unsatisfactory. This was due to the excessive adhesion of spores to the filter material and surfaces of the components employed for constructing the filtration system, originally designed for filtration of *L. pneumophila*^12^. That observation is consistent with the reports of strong adhesion of bacterial spores to hydrophobic surfaces^14,15^.

Furthermore, it has been reported that the electrical polarity of surfaces, as well as spore morphology can affect the adhesion of spores to different solid surfaces^16^. For instance, it has been observed that *B. cereus* spores show high affinity to surfaces of polyethylene of high density (PEHD), Teflon and polyvinyl chloride (PVC), while they exhibit lower adherence to stainless steel, polyamide-6, and minimal adherence to glass^17^. The hydrophobic characteristic of *B. cereus* and *B. thuringiensis* spores is mainly attributed to their outermost layer called exosporium with collagen-like region and the C-terminal domain of a predominantly abundant surface proteoglycan called *Bacillus* collagen like protein A (BclA)^18–21^.

To investigate the pre-concentration of spores intended for biosensing, we have examined PVDF, fiberglass (FG), and polyether sulfone (PES) filters installed in a water sampling module dedicated for collecting spores (WSM-S). The filter with superior performance was employed for processing *Bacillus* spores and evaluating further its repetitive use.

## Materials and methods

### *B. cereus* group strains and reagents

*Bacillus thuringiensis* subspecies (subsp.) *kurstaki* strain HD1 (*B. thuringiensis kurstaki*) derived from Foray 48B, and *Bacillus cereus* ATCC 11,778 obtained from the American Type Culture Collection were used in a spore state for the concentration performance evaluation of WSM-S. The *B. cereus* and *B. thuringiensis* spores were grown for 8 days to sporulate^22^. The sporers were purified by using an aqueous two-phase system comprising polyethylene glycol (PEG) and potassium phosphate with ~ 90% purity for *B. cereus* spores and ~ 93% for *B. thuringiensis* spores, as confirmed by Schaeffer–Fulton staining method and microscopy^23^. Phosphate buffered saline (PBS) solution (10×: 20 mM phosphate-1.4 M NaCl, pH 7.4) used after dilution to 1 × PBS to backwash spores, sodium chloride employed for the preparation of the spores suspensions to be filtered, Luria Bertani broth (LB) and agar were all obtained from Sigma-Aldrich (Ontario, Canada). Hydrophilic polyvinylidene fluoride (PVDF) membranes (0.45 µm, Sigma-Aldrich, ON, Canada), fiber glass (FG) membranes (0.3 µm, Sterilitech, WA, USA) and polyethersulfone (PES) membranes (0.45 µm, Cole Parmer, QC, Canada) were all 47 mm in diameter.

### Water sampling module

**Figure 1:**
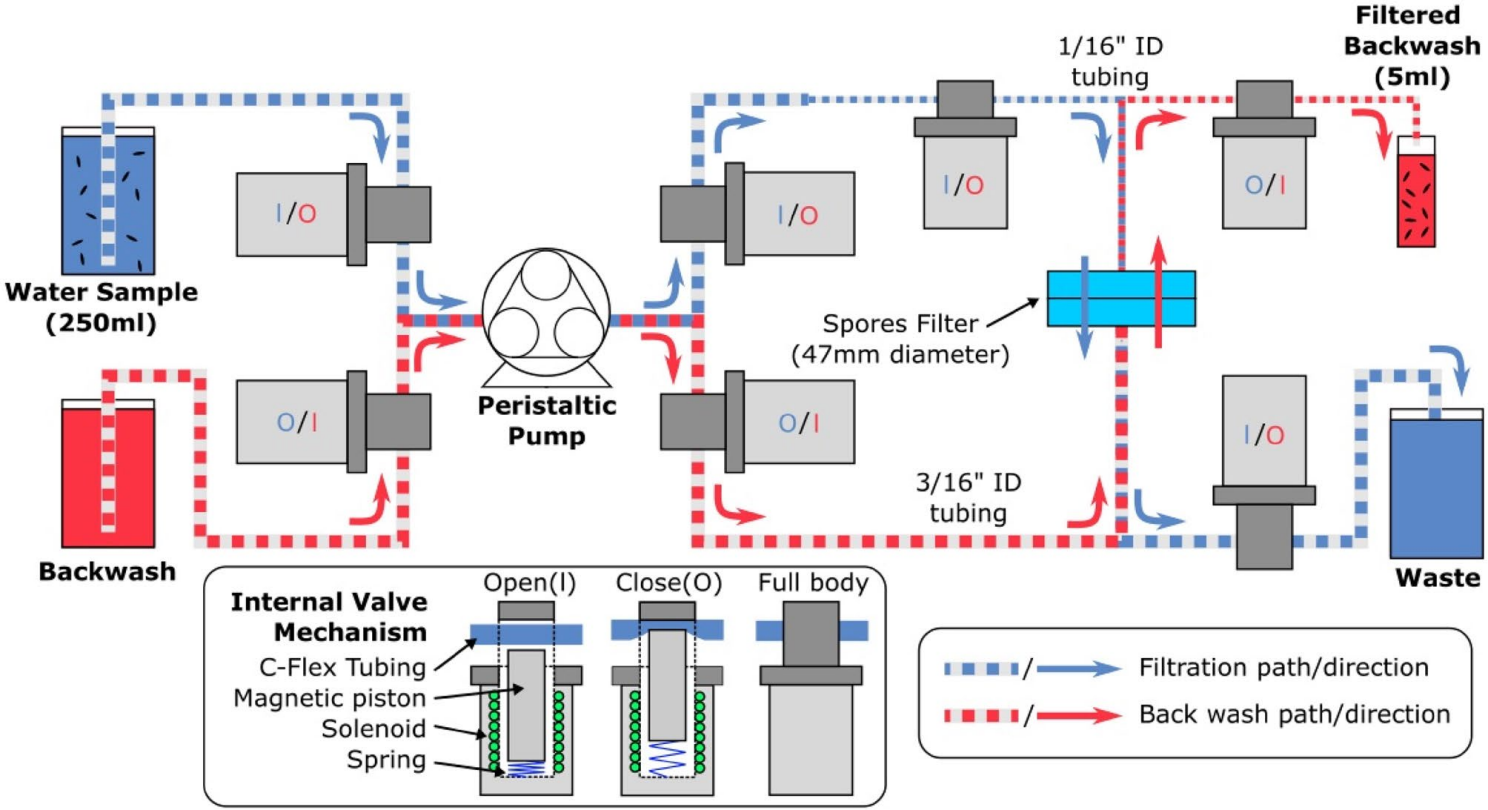
Schematic diagram of the water sampling module (WSM-S) used to filter and concentrate spores into a 5 mL backwash solution from a 250 mL water reservoir. The filtration (blue arrows) and backwash (red arrows) runs are indicated by respective flow directions controlled by the peristaltic pump and a set of electromagnetic pinch valves.

A schematic diagram of the WSM-S unit is shown in Fig. 1. The unit employs a peristaltic pump and a set of pinch valves that allow processing of bacterial suspensions while eliminating contact of metallic or plastic parts of the valves employed for building a conventional WSM^12^. That minimized chemical degradation of the system and eliminated adventitious contamination of processed samples. The C-FLEX type tubing with high biochemical compatibility did not interact with the investigated spores as verified with a series of dedicated experiments (Fig. S1, Supplementary information). The automated process of filtration, backwashing, disinfecting, and cleaning was ensured by an Arduino microcontroller connected to a relay of 12 V sources employed for switching on/off individual valves.

The operation of the system is based on the application of a user-friendly Python coded interface. The first step involves filtration of a pre-determined volume of bacterial suspension in water (250 mL in the current experiments) through either a PVDF, FG or PES filter (blue arrows in Fig. 1). The filtered water is collected in a waste bottle and the spores are retained by the filter membrane. During the second step, the spores retained by the membrane are collected into a Falcon tube by reversing the flow direction and backwashing with 1 × PBS (red arrows in Fig. 1). Only 5 mL of the backwash are collected (monitored with a sensitive photodiode) to avoid excessive dilution of spores. A time sequence of the processing steps executed by the WSM-S unit (cleaning of the system, filter installation, suction of spore suspension, and backwashing) is schematically shown in Supplementary information (Fig. S2).

### Filtration of *B*. *thuringiensis kurstaki* and *B. cereus* spores

Initial suspensions were prepared by spiking saline water (9 g/L NaCl) with investigated spores. The suspensions were circulated through the WSM-S module equipped with either a PVDF, FG or PES filter placed in the holder. To determine the initial concentration, 100 μL of samples were taken from *B. thuringiensis kurstaki* or *B. cereus* suspensions and seeded on LB-agar. Following a 24-h growth period at 35 °C, the concentration was determined by the plate count method. From a tenfold diluted backwash (BW), several 100 μL samples were seeded in LB agar and the numbers of grown colonies were counted after a 24-h growth period at 35 °C.

Before each filtration run, the module was decontaminated by incubating with a 1% bleach solution for 60 min. The system was then rinsed by 3 successive washes using hot water (~ 80 °C), and 3 times with room-temperature deionized (DI) water. The filtration performance was evaluated by calculating the concentration factor (CF) acording the following formula:

$$\mathrm{CF }= \frac{{\text{Spore concentration in the backwash suspension}}}{{\text{Spore concentration in the filtered suspension}}}$$

Under ideal conditions, where 100% of spores were collected from a 250 mL sample of suspension, the maximum concentration factor (CF_max_), calculated as the ratio of the initial to the final volume, is 50×.

To evaluate the adherence of *B. cereus* spores to FG and PES filters, the after-backwashing filters were ground in 50 mL of 1 × PBS using a bleach-sterilized high-speed hand blender (Cuisinart Smart Stick Two-Speed Hand Blender—CSB-75BKC). 200 µL of the resulting filters micro pulp suspensions were used to inoculate three LB agar plates and incubated for 24 h at 35 °C. The number of colonies present helped determine the adherence of *B. cereus* spores to the filter material. In the second experiment, after backwashing FG, PES or PVDF filters employed for filtration of the *B. cereus* suspension at initial concentration of 1 × 10^2^ spores/mL, the filters were placed in a Petri dish with LB agar, making sure that all the filter surface remained in contact with the medium. The filters were removed after 1 min, and the growth of *B. cereus* was evaluated after 24-h incubation at 35 °C.

### Reusability of FG filters

Repetitive runs with FG filters were carried out for up to 6 consecutive filtrations of *B. thuringiensis kurstaki* spore suspensions. Each filtration run consisted of the following steps: (1) Flow of 250 mL of a saline water suspension of *B. thuringiensis kurstaki* spores, (2) Production of 5 mL of backwashed suspension in 1 × PBS, (3) 1 h exposure of the WSM-S system to 1% Bleach, (4) 3-times washing in hot (80 °C) DI water, and (5) 3-times washing with DI water at room temperature. The steps were repeated with the same filter, and 100 μL of filtrate collected after each filtration run were used to seed LB-agar to enumerate spores not captured by the filter.

### Confocal microscopy measurements

The morphology of FG filters was investigated with a confocal microscope (KEYENCE model VK-X1100). The instrument works with a 404 nm laser operating in the reflection mode. The images collected with a 150× lens had a maximum examination depth of 150 µm, and 9375 images collected with a 0.016 µm step were used for the 3D reconstruction. The surface area of 92.1 µm × 72.8 µm was scanned with a 0.13 µm resolution, 1.6 nm vertical resolution and the overall magnification of 3600×. The average roughness (Sa), defined as a mean difference in height of each point compared to the arithmetical mean of the surface, was used to characterize the texture of the filter.

### Concentrating *B. thuringiensis kurstaki* spores from cooling tower water samples

Samples of cooling tower water (CTW) were acquired from one of the cooling towers of the Université de Sherbrooke. The OD_600nm_ of CTW samples was at 0.01 compared to "zero" of a blank saline water sample. The CTW samples were inoculated with *B. thuringiensis kurstaki* spores to produce a suspension at 2.75 × 10^2^ spores/mL.

Four filtration runs, each of a 250 mL suspension, were completed with WSM-S using a FG filter and 5-mL backwashes were produced each time. Between each filtration run, the WSM-S system was washed only with DI water. The samples were heated for 10 min at 80 °C to inactivate the CTW flora. The samples prepared in this way were employed to inoculate LB-agar and the results of the cultivation at 35 °C were analyzed after the standard incubation period of 24 h.

## Results

### Concentration factors for *B. thuringiensis kurstaki* and *B. cereus* spores using different types of filters

**Figure 2:**
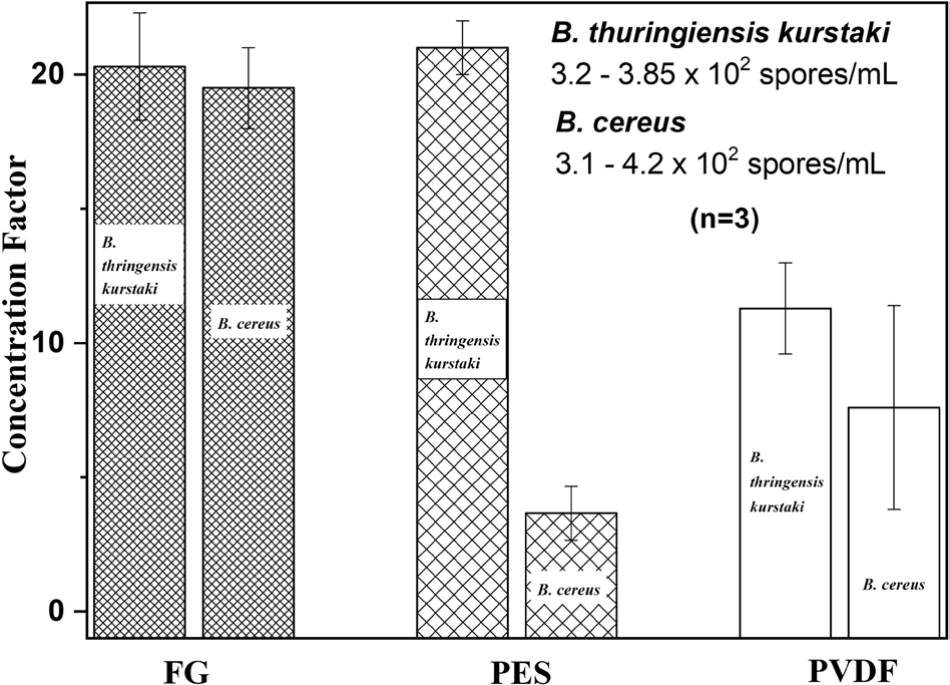
Concentration factors of *B. thuringiensis kurstaki* and *B. cereus* spores obtained with FG, PES, and PVDF filters for suspensions at initial concentration of 3.14.2 × 10^2^ spores/mL. The data bars represent standard errors of the mean from three separate experiments.

The CF values achieved for *B. thuringiensis kurstaki* and *B. cereus* spores with FG, PES, and PVDF filters are presented in Fig. 2. CFs of ~ 20 were obtained with the FG filter for both types of spores. The PES filter provided CF of ~ 21 and ~ 4 for *B. thuringiensis kurstaki* and *B. cereus* spores, while CF values of ~ 11 and ~ 7 were obtained with the PVDF filter for *B. thuringiensis kurstaki* and *B. cereus* spores. Thus, FG and PES filters concentrate *B. thuringiensis kurstaki* spores by the same order of magnitude, but only the FG filter concentrates *B. cereus* spores better than the other two filters.

**Figure 3:**
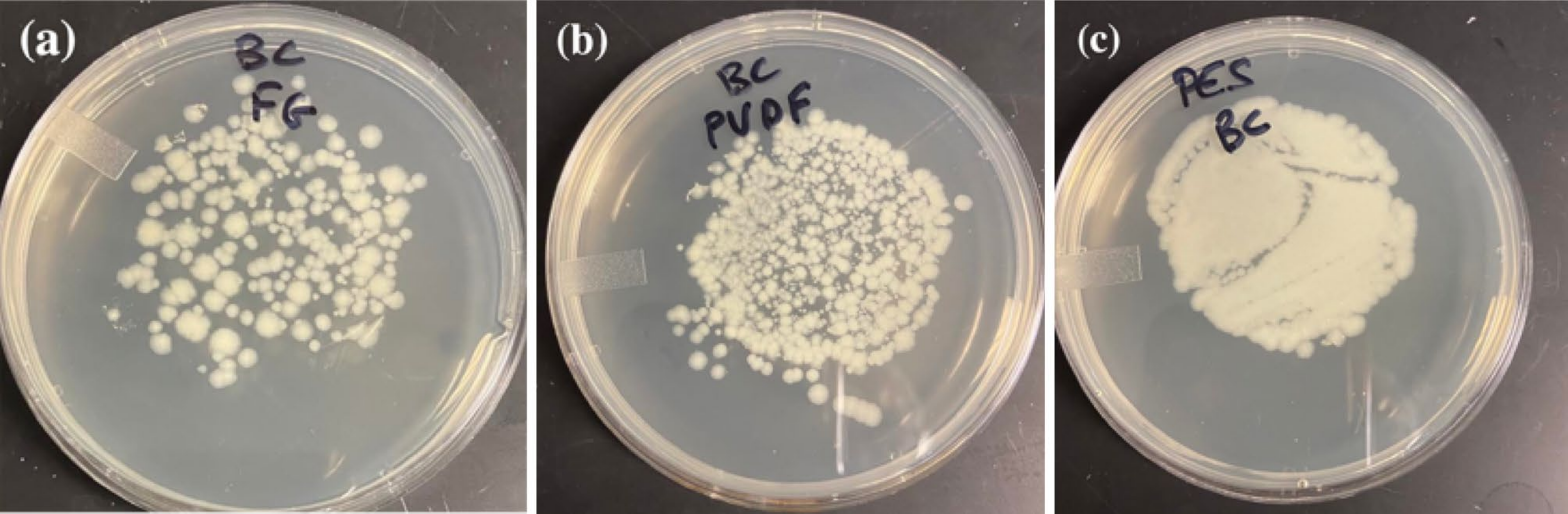
Images of stamp-grown *B. cereus* spores from backwashed FG (**a**), PVDF (**b**) and PES (**c**) filters used to retain spores from 250 mL suspensions at 1 × 10^2^ spores/mL.

Following the filtration of the suspension containing *B. cereus* spores, the WSM-S containing each filter type was backwashed and LB-agar was stamped with the filters. The resulting growth indicated that lower numbers of *B. cereus* spores were retained on the FG filter while most of the spores were retained by the PES filter (Fig. 3).

The number of *B. cereus* spores enumerated in suspensions produced by ground FG and PES filters are presented in Table 2.

**Table 2:** Number of *B. cereus* spores remaining on FG and PES filters following the backwashing procedure.

Filter	Initial concentration (spores/mL)	Total spores (in 250 mL)	Spores on filter (n = 3)	% residual spores (n = 3)
FG	1.56 × 10^2^	39,000	9333 ± 2516	~ 26
PES	2.6 × 10^2^	65,000	43,833 ± 1527	~ 68

This experiment demonstrates that ~ 26% and 68% of the initial concentrations of *B. cereus* spores remain on the FG and PES filters, respectively, which is consistent with the greater efficiency of pre-concentrating spores with the FG filters.

### Concentration factors for FG filters collecting ***B. thuringiensis kurstaki*** and ***B. cereus*** spores from low concentration suspensions (N ≤ 3.2 × 10^2^ spores/mL)

Further investigation of the WSM-S performance was carried out using low concentration (10–3.2 × 10^2^ spores/mL) suspensions of *B. thuringiensis kurstaki* or *B. cereus*. Table 3 shows the CFs values of 22.5 ± 1.5 and 19.5 ± 1 for *B. cereus* at 10 and 3.1 × 10^2^ spores/mL, respectively. For *B. thuringiensis kurstaki* spores at initial concentrations of 5 × 10^1^, 2 × 10^2^, and 3.2 × 10^2^ spores/mL, the CF values were 23.8 ± 3.4, 22 ± 1.5 and 20.3 ± 2, respectively. Thus, the CFs values appear independent of the initial concentrations for the investigated concentration range.

**Table 3:** Concentration factors of *B. thuringiensis kurstaki* and *B. cereus* spores for weakly contaminated saline water suspensions obtained with the FG filter.

Initial concentration (spores/mL)	CF obtained for *B. cereus* spores	CF obtained for *B. thuringiensis kurstaki* spores
10	22.5 ± 1.5	–
5 × 10	–	23.8 ± 3.4
2 × 10^2^	–	22 ± 1.5
3.1 × 10^2^	19.5 ± 1	–
3.2 × 10^2^	–	20.3 ± 2

### Reusability of FG filters

To investigate the reusability of FG filters, 8 consecutive filtrations of *B. thuringiensis kurstaki* spores suspensions (4.3 × 10^2^ spores/mL) were carried out using the same filter. In this experiment no bleach treatment was used between filtration runs. Spore concentrations in the backwash and in the filtrate were determined by plate counting and CFs were calculated for each filtration run (Table 4). CF values between 21.7 ± 0.5 and 18 ± 0.5 were achieved for the first 6 filtration runs but a significant drop in CF was observed for the 7th and 8th filtrations (14 ± 2.2 and 8.9 ± 1.8, Table 4). The loss of the filter’s ability to retain spores for more than 6 runs was confirmed by the presence of spores in the 7th and 8th filtrates.

**Table 4:** Concentration factors of *B. thuringiensis kurstaki* spores achieved for backwashing of the same FG filter and concentration of spores in filtrates obtained with bleach-free filtration runs (The limit of detection at 10 spores/mL for 100 µL samples).

Filtration run *B. thuringiensis kurstaki* (4.2–4.3 × 10^2^ spores/mL)	CF	Second BW concentration (spores/mL)	Filtrate concentration (spores/mL)
1	20.7 ± 0.8	No growth	No growth
2	20.6 ± 2.2	No growth	No growth
3	20.3 ± 0.25	No growth	No growth
4	21.7 ± 0.5	No growth	No growth
5	19.5 ± 1	No growth	No growth
6	18 ± 0.5	No growth	No growth
7	14 ± 2.2	No growth	159 ± 33
8	8.9 ± 1.8	No growth	191 ± 32

The effect of exposing FG filters to 60 min bleaching was investigated with repeated filtration runs using suspension at 2.5 × 10^2^ spores/mL. The results illustrate that bleaching had a negligible effect on the CF values for up to 3 filtration runs, which suggests that the structural integrity of an FG filter is maintained under these conditions (Table 5). It is possible that a shorter than 1 h exposure of the filters to bleach may also play a role in the longer-term maintenance of their structural integrity, but this effect was not investigated in the current experiment.

**Table 5:** Concentration factors of *B. thuringiensis kurstaki* spores achieved for backwashing and bleaching FG filters reused for up 6 times.

Filtration run *B. thuringiensis kurstaki* (2.5 × 10^2^ spores/mL)	FG filter 1 CF	FG filter 2 CF	FG filter 3 CF	FG filter 4 CF
1	23.5	20.3	19.2	25.0
2	24.0	22.0	19.0	21.4
3	20.0	20.2	22.0	22.1
4	13.0	20.0	20.0	22.0
5	–	19.0	16.0	17.0
6	–	14.1	10.0	15.4

**Figure 4:**
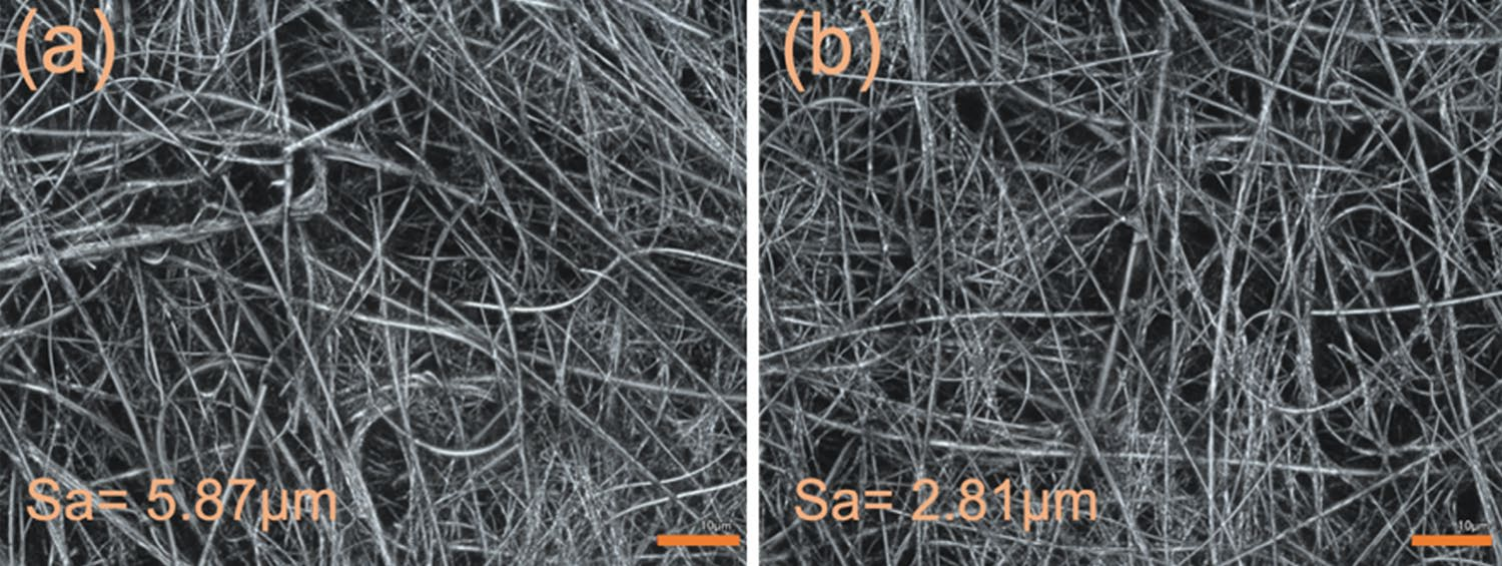
Confocal microscopy images of (**a**) a fresh FG filter, and (**b**) a FG filter after 3 filtration runs with backwashing and bleach decontamination. The scale bar corresponds to 10 μm.

Representative confocal microscopy micrographs of a fresh filter and of a filter used for 3 consecutive filtration runs with bleaching applied after each backwashing step are shown in Fig. 4. The roughness of the filters is illustrated by the decrease of the Sa value from 5.87 µm (fresh filter) to 2.81 µm (reused filter). A significant drop in the Sa value to 4.12 µm was observed for the filter backwashed and bleached only once (Table 6). The reuse of filters further reduced their Sa values to 2.4–2.9 µm, without a significant difference observed for 3-times or 6-times re-used filters (Table 6). Ultimately, filtration of a *B. thuringiensis kurstaki* spore suspension at 1 × 10^6^ spores/mL without backwashing produced a filter with Sa = 0.94 µm (see Supplementary information for examples of confocal microscopy images of the related filters, Fig. S3).

**Table 6:** Sa values for FG filters used for the indicated number of successive filtrations including backwashing and bleaching.

Filter	Applied procedure	Sa (µm)
FG	Fresh filter	5.87
FG1^++^	Used once, backwashed and bleached	4.12
FG 3^+^	Used 3 times, backwashed and bleached after 1st and 2nd filtrations, and backwashed after 3rd filtration	2.91
FG 3^++^	Used 3 times, backwashed and bleached after each run	2.81
FG 3	Used 3 times, backwashed and bleached after 1st and 2nd filtration	2.44
FG 6^+^	Used 6 times, backwashed and bleached after 1–5 filtrations, and backwashed after 6th filtration	2.79
FG^HCBTK^	Filter used with highly concentrated suspension of *B. thuringiensis kurstaki* spores (10^6^ spores/mL), not backwashed	0.94

The (+) and (++) indicate if either backwashing only, or backwashing and bleaching were applied, respectively, as the last steps before collecting confocal images. HCBTK means “highly concentrated suspension of *B. thuringiensis kurstaki* spores”.

Culture analysis showed that repeated filtrations (up to 4 times) of 3 × 10^3^ *B. thuringiensis kurstaki* spores/mL generated filtrates free of spores. However, 8 × 10^1^ and 7.5 × 10^2^ spores/mL were detected in the filtrates produced with the 5th and 6th filtrations, respectively, which constitutes 2.6 and 25% of the original spore suspension (see Fig. S4). These results are consistent with the significantly reduced CF values observed for the 5th and 6th filtration runs (Table 5). The repetitive collection of *B. thuringiensis kurstaki* spores suspended in CTW using the same FG filter re-used up to 4 times (washed with DI water between each filtration step) revealed that the CF decreased from 18.9 to 16.7 (Supplementary information, Table S1).

## Discussion

The experiments involving PVDF, FG, and PES filters for collecting *B. thuringiensis kurstaki* and *B. cereus* spores revealed the superior performance of the FG filter delivering CF ≅ 20 for both types of spores. The performance of a filter is of particular importance to the backwashing step, and dependant on the electrostatic interaction between hydrophobic *Bacillus* strains and membranes of the investigated filters. For instance, zeta (ζ)‐potential of the glass surface is equal to − 50 mV at *p*H = 7^24^, thus the repulsive interactions of the hydrophilic FG filter with hydrophobic spores is expected to facilitate the release of spores during the backwashing step^17,25^. This compares to the reduced performance of PES filters whose material is characterized by ζ‐potential of − 20 mV in a comparable *p*H environment^26^. Furthermore, the poorest filtration performance of PVDF filters observed in our experiments (Fig. 2) must be related to the augmented hydrophobicity of the filter material characterized by ζ‐potential of + 35 mV. However, other authors have reported that the ζ‐potential of PVDF filters grafted with hydrophilic molecules, such as lysine or sulfone, could become as high as − 50 mV^27^. The analysis of used filters revealed that some spores remained on the filter material after backwashing (Fig. 3), which led to reduced CF values for all the investigated filters. The repetitive use of a filter could lead to further reduced CFs, as observed with the 4th filtration run involving the bleaching step included in the process (Table 4).

The adhesion of spores to different surfaces has been the subject of extensive investigations. For instance, the extended Derjaguih, Landau, Verwey and Overbeek (XDVLO) theory and environmental scanning electron microscopy (ESEM) were used to predict and experimentally verify the attachment of four *Penicillium* strains to cedar wood^28^. The model was corroborated by data concerning a specific *Penicillium* strain, but did not fit the other three strains investigated by those authors^28^. That illustrates the complexity of the phenomena related to the microbial spore interactions with different surfaces.

The results discussed in this report suggest that filtration of *B. cereus* spores that leads to CF = 20 with FG filters reused up to 5 times is attractive for supporting the operation of a semi-automated workstation designed for monitoring environmental or agricultural water environments. We note that a CF = 50 would correspond to the perfect filtration of a 250 mL suspension and collecting all the spores in a 5 mL volume of the backwash material. Such a result seems unrealistic in view of the studies of cake backwashing from membrane filters that illustrated a dependence of the process on parameters such as turbulence, backwash duration, as well as flow speed and pressure^29–31^. Given the reasonable reproducibility of the backwashing conditions achieved with our WSM-S unit, the difference in CF values obtained in our experiments with different filters is mainly related to the weaker adherence of the *B. thuringiensis kurstaki* spores to the FG filter material ^17^. The evaluation of a trapping efficiency of *B. cereus* spores after backwashing showed that 68% of the filtered spores were still attached to the PES filter compared to 26% of these spores attached to the FG (Table 2). That result confirms that *B. cereus* spores interact strongly with the PES filter, which is consistent with the lower CF values observed for this filter.

## Conclusions

A filtration module (WSM-S) dedicated for collecting water samples containing spores from members of the *Bacillus cereus* group was designed and evaluated for the potential application as a quasi-autonomously operating tool. The application of pinch valves eliminated the risk of cross-contamination due to the potential reaction between materials commonly used in construction of electromagnetic valves and sampled liquids. The backwashing of PES and PVDF filters revealed the difficulty in releasing spores trapped by those filters, while FG filters were found efficient for the repetitive collection of spores. The structural integrity of FG filters investigated with confocal microscopy revealed a reduction of the filter porosity indicated by the Sa diminution from 5.87 µm (fresh filter) to 2.81 µm (3 times used filter). The 20× concentration factor of *B. thuringiensis kurstaki* spores obtained with the FG filter and reproduced with 4 consecutive runs (backwashed and bleached between each run) suggests attractive application of such a filter for the operation of a semi-automated water sampling module. We argue that a greater number of repetitive filtrations runs using similar filters should be possible with the further advanced knowledge of materials and the advancement of backwashing protocols. This should lead towards practical advancement of the concept of a quasi-autonomous biosensing workstation for monitoring the presence of bacteria and spores in the environmental and anthropogenic water.

## Supplementary Information

Supplementary Information.

## Data Availability

The datasets used and/or analyzed during the current study available from the corresponding author (JJD) on request.
