# Supplementary material for: Semi-automated water sampling module for repeated sampling and concentration of Bacillus cereus group spores
Source: Sci Rep. 2023 Jan 16;13:831. doi: 10.1038/s41598-023-27900-0 (PMC9842714; doi:10.1038/s41598-023-27900-0)
Supplement: Supplementary file 1 — Supplementary Information. [file 41598_2023_27900_MOESM1_ESM.pdf]

Supplementary information

Semi-automated water sampling module for repeated sampling and concentration of *Bacillus cereus* group spores

Walid M. Hassen[1], Jonathan Vermette[1], Houman Moteshareie[1,2], Azam F. Tayabali[1,2*], Jan J. Dubowski[1*]

[1]Interdisciplinary Institute for Technological Innovation (3IT), CNRS IRL-3463, Laboratory for Quantum Semiconductors and Photon-based BioNanotechnology, Department of Electrical and Computer Engineering, Université de Sherbrooke, 3000, boul. de l'Université, Sherbrooke, Québec J1K 0A5, Canada.
[2] Biotechnology Laboratory, Environmental Health Science and Research Bureau, Healthy Environments and Consumer Safety Branch, Health Canada, Ottawa, Ontario, Canada.

[*]Address correspondence to

Jan.J.Dubowski@usherbrooke.ca (submitting author)

Azam.Tayabali@Canada.ca

Figure S1. Schematic illustration of the protocol performed by the WSM-S unit: (a,b) cleaning of the system, c) filter installation, d) intake of water and filtration, e) backwashing.

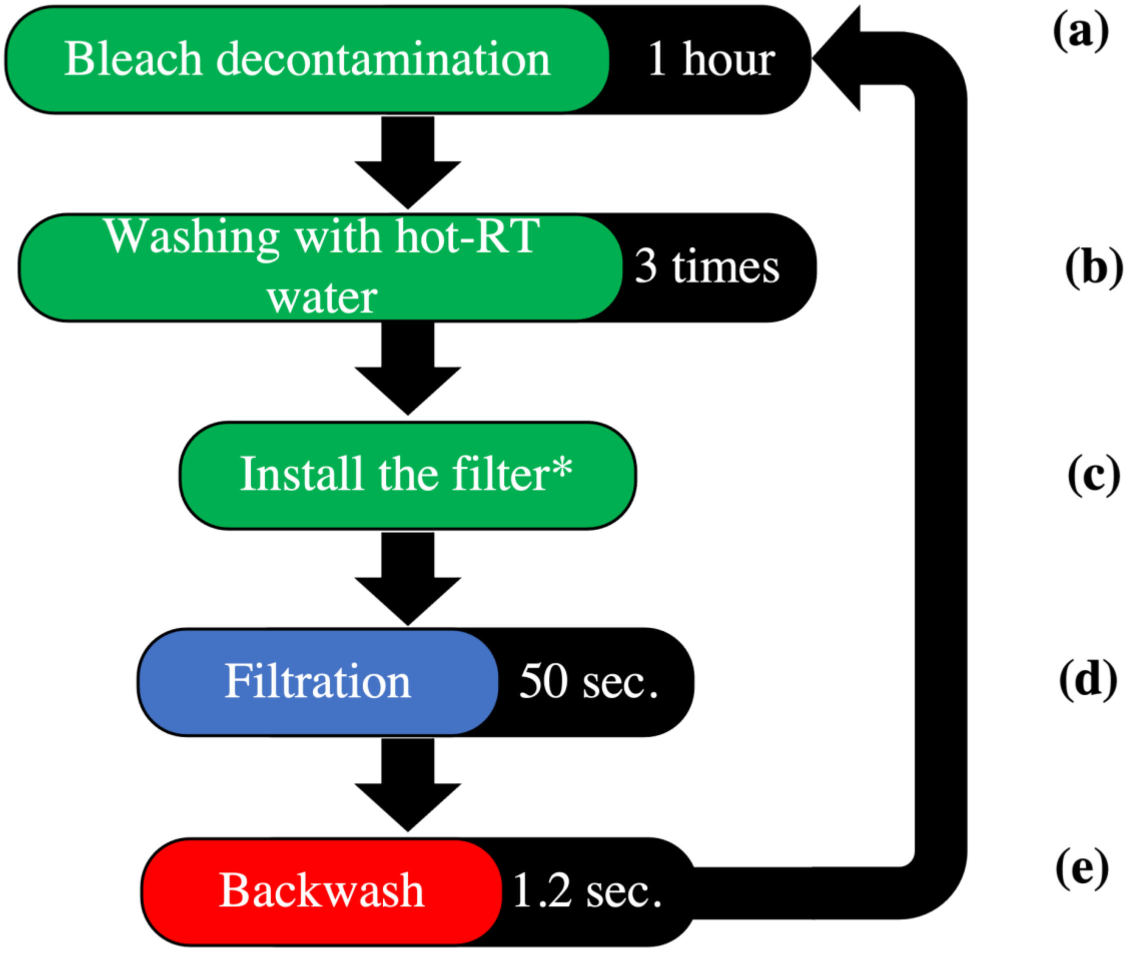

Figure S2. Confocal microscopy images of FG filters at different stage of reusability where $(^{+})$ indicates a backwashed filter, $(^{+})$ absent indicates a non-backwashed filter, $(^{++})$ indicated a filter backwashed and bleached. The index number corresponds to the number of filtrations. FG^{HCBTK} corresponds to a filter used to retain highly concentrated suspension of Btk spores without backwashing. The scale bar corresponds to 10 µm.

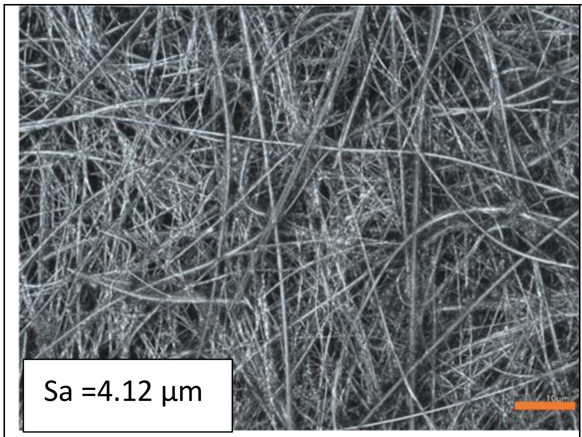

FG 1^{++}

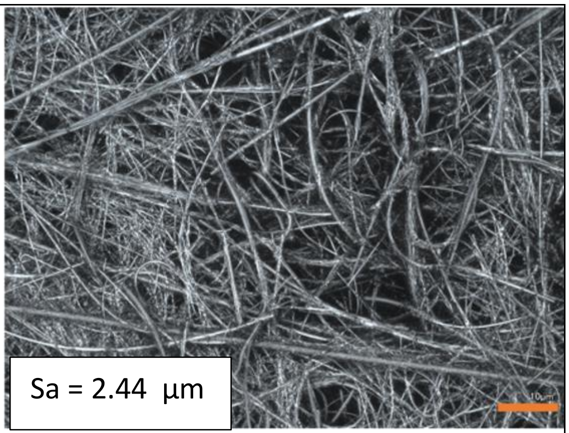

FG 3

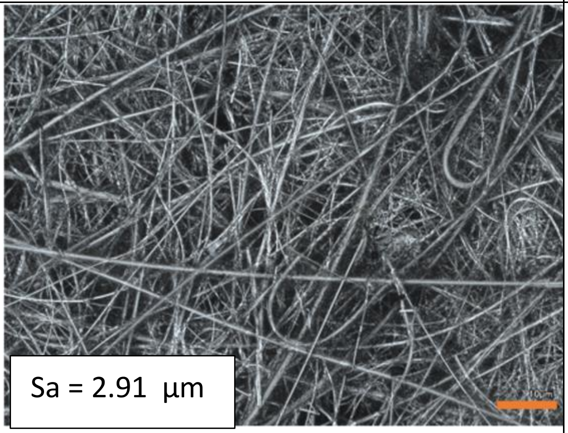

FG 3^{+}

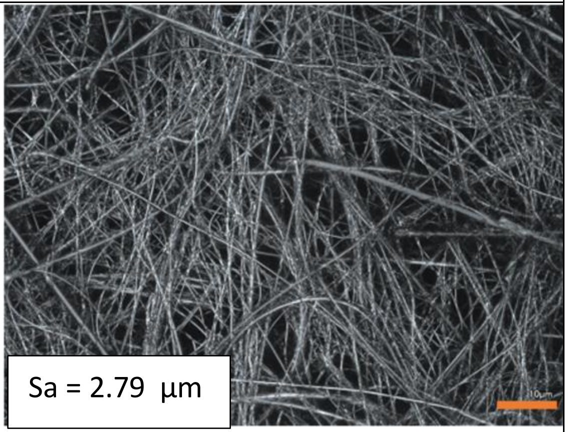

FG 6^{+}

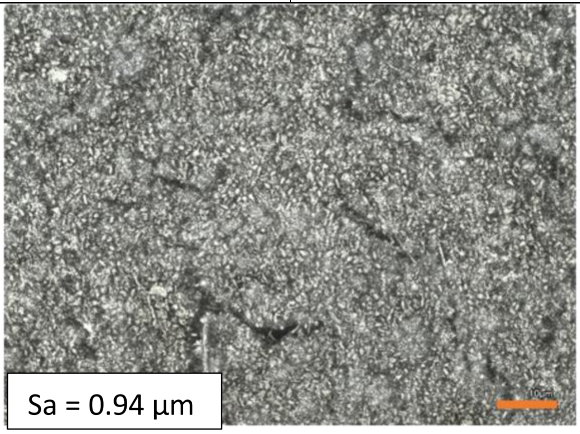

FG^{HCBTK}

Figure S3. Petri dish images following a 24-hours growth at 35ºC of the filtrate generated following up to 6 filtration runs of *Bacillus thuringiensis* subs. *kurstaki* suspensions at 3000 CFU/mL using the same FG filter (bleach disinfected between each filtration).

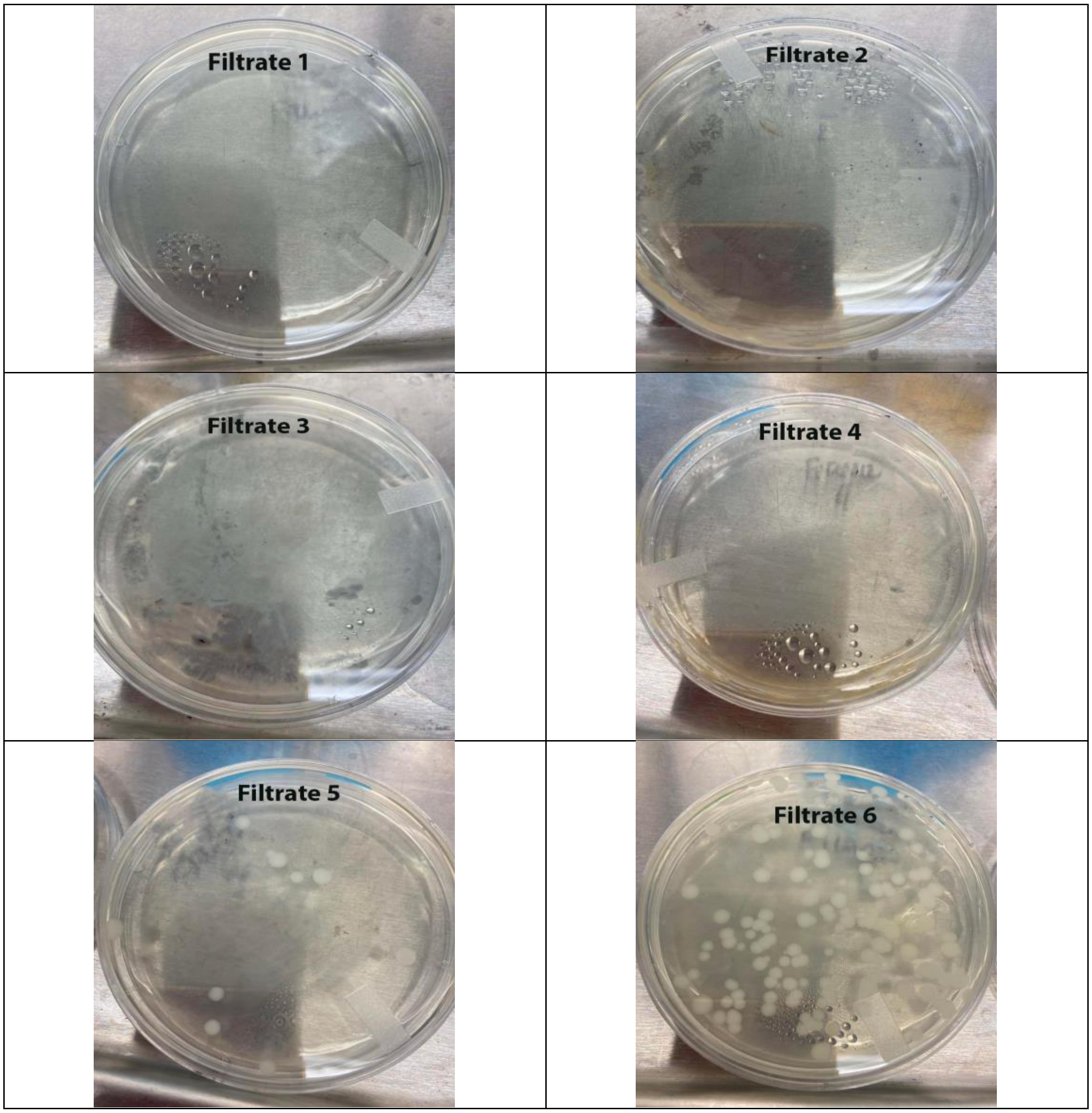

Table S1. Concentration factors obtained for *Bacillus thuringiensis* subs. *kurstaki* suspensions in cooling tower water with the same FG filter reused up to 4 times (washed with DI water between each filtration).

	**CF**
Filtration 1	18.9
Filtration 2	17.8
Filtration 3	17.5
Filtration 4	16.7

Concentrating Btk spores from CTW samples. As we can see in Table S1, CF ~ 17.5 is obtained for 4 consecutive filtration runs of Btk spore suspensions at 275 CFU/mL in CTW and backwashing.
